# Crystal structure of bis­(tetra­phenyl­phospho­nium) bis­(cyanido-κ*C*)(29*H*,31*H*-tetra­benzo[*b*,*g*,*l*,*q*]porphinato-κ^4^ *N* ^29^,*N* ^30^,*N* ^31^,*N* ^32^)ferrate(II) acetone disolvate

**DOI:** 10.1107/S2056989015001735

**Published:** 2015-01-31

**Authors:** Miki Nishi, Masaki Matsuda, Norihisa Hoshino, Tomoyuki Akutagawa

**Affiliations:** a Department of Chemistry, Kumamoto University, Kurokami 2-39-1, Chuo-ku, Kumamoto 860-8555, Japan; b Institute of Multidisciplinary Research for Advanced Materials, Tohoku University, Katahira 2-1-1, Aoba-ku, Sendai 980-8577, Japan

**Keywords:** crystal structure, tetra­benzoporphine, iron(II) complex, C—H⋯N inter­actions, C—H⋯π inter­actions

## Abstract

The crystal structure of the title compound, (C_24_H_20_P)_2_[Fe(C_36_H_20_N_4_)(CN)_2_]·2C_3_H_6_O, is constructed from a tetra­hedral Ph_4_P^+^ (tetra­phenyl­phospho­nium) cation, one [Fe(tbp)(CN)_2_]^2−^ anion (tbp = tetra­benzoporphyrin in its doubly deprotonated form), located on a centre of inversion, and an acetone mol­ecule as crystallization solvent. Since the mol­ecular structure of the *M*(tbp) moiety is insensitive to the kind of metal ion and its oxidation state, bond lengths and angles in the [Fe(tbp)(CN)_2_]^2−^ anion are similar to those in other *M*(tbp) compounds. The Fe^2+^ ion, located on a centre of inversion, is coordinated by four N atoms of tpb in the equatorial plane and by two C atoms of the cyanide anion at axial positions in a slightly distorted octa­hedral configuration. The packing is stabilized by C—H⋯N inter­actions between the Ph_4_P^+^ cation and the CN^−^ ligand of the [Fe(tbp)(CN)_2_]^2−^ anion, and by C—H⋯π inter­actions between the Ph_4_P^+^ cation, acetone solvent mol­ecules and the [Fe(tbp)(CN)_2_]^2−^ anion.

## Related literature   

For [Fe(tbp)(CN)_2_]^−^ and [Co(tbp)(CN)_2_]^−^ complexes, see: Matsuda *et al.* (2011 ▸, 2014 ▸); Nishi *et al.* (2015 ▸). The crystal structure of metal-free tbp was reported by Aramaki & Mizuguchi (2003 ▸).
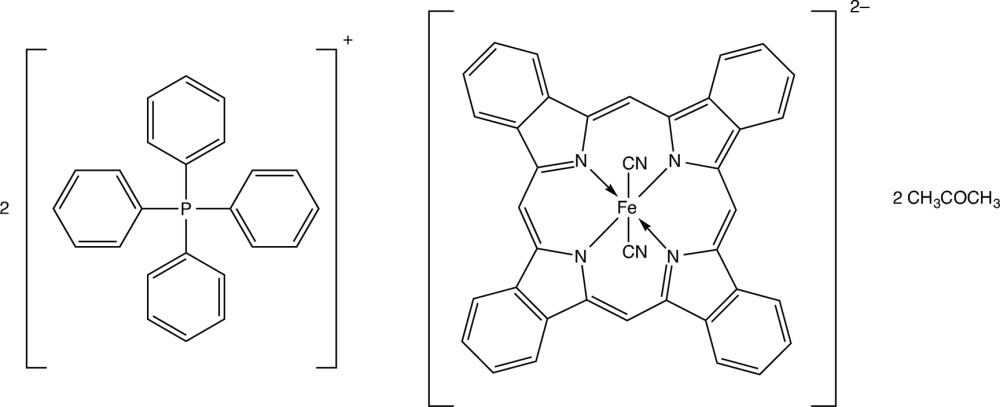


## Experimental   

### Crystal data  

(C_24_H_20_P)_2_[Fe(C_36_H_20_N_4_)(CN)_2_]·2C_3_H_6_O
*M* *_r_* = 1411.35
Triclinic, 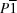

*a* = 11.8412 (3) Å
*b* = 12.7764 (3) Å
*c* = 13.8298 (3) Å
α = 67.217 (1)°
β = 66.915 (1)°
γ = 85.510 (1)°
*V* = 1767.88 (7) Å^3^
*Z* = 1
Cu *K*α radiation
μ = 2.59 mm^−1^
*T* = 120 K
0.20 × 0.10 × 0.05 mm

### Data collection  

Rigaku R-AXIS RAPID diffractometer
Absorption correction: multi-scan (*ABSCOR*; Higashi, 2001 ▸) *T*_min_ = 0.764, *T*_max_ = 1.000
20279 measured reflections
6348 independent reflections
3518 reflections with *I* > 2σ(*I*)
*R*_int_ = 0.065

### Refinement  

*R*[*F* ^2^ > 2σ(*F* ^2^)] = 0.068
*wR*(*F* ^2^) = 0.198
*S* = 0.98
6348 reflections
469 parameters
H-atom parameters constrained
Δρ_max_ = 0.71 e Å^−3^
Δρ_min_ = −0.36 e Å^−3^

### 

Data collection: *RAPID-AUTO* (Rigaku, 1999 ▸); cell refinement: *RAPID-AUTO*; data reduction: *RAPID-AUTO*; program(s) used to solve structure: *SHELXS97* (Sheldrick, 2008 ▸); program(s) used to refine structure: *SHELXL97* (Sheldrick, 2008 ▸); molecular graphics: *ORTEP-3 for Windows* (Farrugia, 2012 ▸) and *Mercury* (Macrae *et al.*, 2006 ▸); software used to prepare material for publication: *Yadokari-XG* (Wakita, 2001 ▸) and *publCIF* (Westrip, 2010 ▸).

## Supplementary Material

Crystal structure: contains datablock(s) I, New_Global_Publ_Block. DOI: 10.1107/S2056989015001735/wm5120sup1.cif

Structure factors: contains datablock(s) I. DOI: 10.1107/S2056989015001735/wm5120Isup2.hkl

**Fig. 1.**
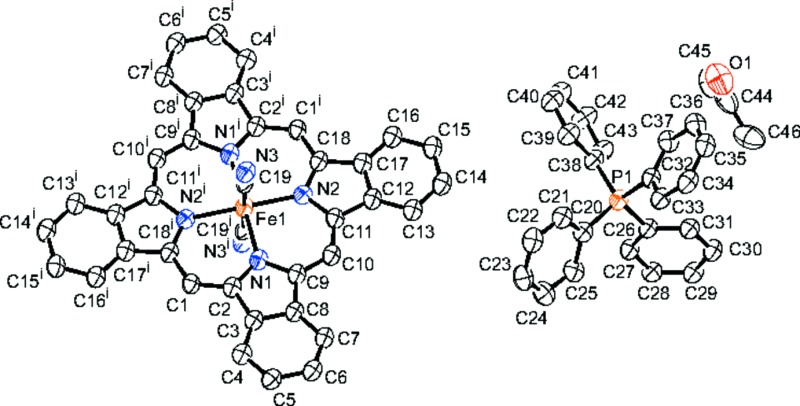
The Ph4P+, [Fe(tbp)(CN)2]2- and acetone molecular units in the title compound. Displacement ellipsoids are drawn at the 30% probability level. [Symmetry code: i) -x, -y, -z.]

4 + 2 2− x y z . DOI: 10.1107/S2056989015001735/wm5120fig1.tif
The Ph_4_P^+^, [Fe(tbp)(CN)_2_]^2−^ and acetone mol­ecular units in the title compound. Displacement ellipsoids are drawn at the 30% probability level. [Symmetry code: i) −*x*, −*y*, −*z*.]

**Fig. 2.**
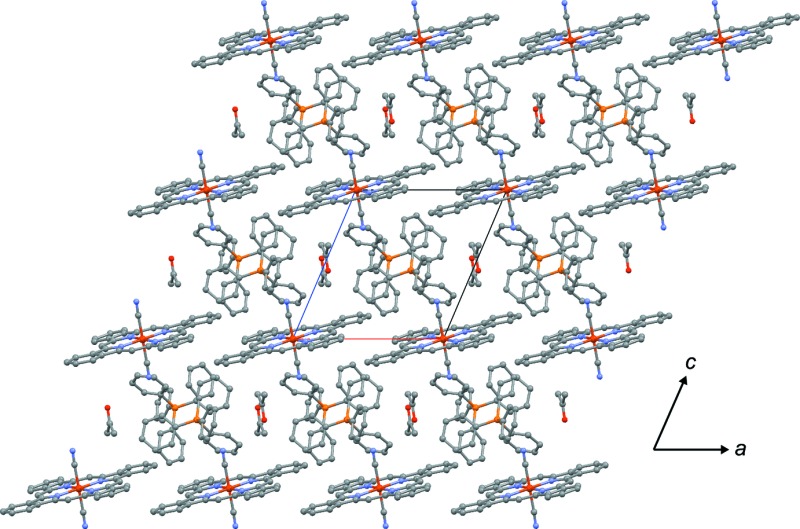
The packing of the title compound viewed down [010].

. DOI: 10.1107/S2056989015001735/wm5120fig2.tif
The packing of the title compound viewed down [010].

CCDC reference: 1045739

Additional supporting information:  crystallographic information; 3D view; checkCIF report

## Figures and Tables

**Table 1:** Hydrogen-bond geometry (, ) *Cg*1, *Cg*2, *Cg*3 and *Cg*4 are the centroids of the N2/C11/C12/C17/C18, C12C17, C26C31 and N1/C2/C3/C8/C9 rings, respectively.

*D*H*A*	*D*H	H*A*	*D* *A*	*D*H*A*
C34H22N3^i^	0.95	2.57	3.292(7)	133
C41H28N3^ii^	0.95	2.45	3.181(7)	134
C28H17*Cg*1^iii^	0.95	2.63	3.446(4)	145
C29H18*Cg*2^iii^	0.95	2.76	3.582(4)	145
C33H21*Cg*3^iv^	0.95	2.90	3.729(5)	147
C35H23*Cg*4^i^	0.95	2.92	3.733(4)	145
C45H32*Cg*2^v^	0.98	2.99	3.798(6)	141
C45H33*Cg*1^v^	0.98	2.87	3.469(5)	120

Symmetry codes: (i) 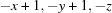; (ii) 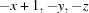; (iii) 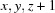; (iv) 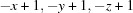; (v) 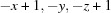.

## supplementary crystallographic information

### S1. Synthesis and crystallization

Fe(tbp) and (Ph_4_P)[Fe(tbp)(CN)_2_] were synthesized by the procedure reported by Nishi *et al.* (2015). Electroreduction of (Ph_4_P)[Fe(tbp)(CN)_2_] in acetone gave black single crystals of the title compound (Ph_4_P)_2_[Fe(tbp)(CN)_2_].

### S2. Refinement

All hydrogen atoms were positioned geometrically with C—H = 0.95 Å for aromatic and C—H = 0.98 Å for methyl, and they were constrained to ride on their parent atoms with *U*_iso_(H) = 1.2*U*_eq_(C) for aromatic and *U*_iso_(H) = 1.5 *U*_eq_(C) for methyl. Reflection 6 12 0 was obstructed from the beam stop and was omitted from the refinement.

### Crystal data

**Table:**

(C_24_H_20_P)_2_[Fe(C_36_H_20_N_4_)(CN)_2_]·2C_3_H_6_O	*Z* = 1
*M**_r_* = 1411.35	*F*(000) = 738
Triclinic, *P*1	*D*_x_ = 1.326 Mg m^−^^3^
*a* = 11.8412 (3) Å	Cu *K*α radiation, λ = 1.54187 Å
*b* = 12.7764 (3) Å	Cell parameters from 9659 reflections
*c* = 13.8298 (3) Å	θ = 3.8–68.1°
α = 67.217 (1)°	µ = 2.59 mm^−^^1^
β = 66.915 (1)°	*T* = 120 K
γ = 85.510 (1)°	Block, black
*V* = 1767.88 (7) Å^3^	0.20 × 0.10 × 0.05 mm

### Data collection

**Table:**

Rigaku R-AXIS RAPID diffractometer	6348 independent reflections
Radiation source: fine-focus sealed tube	3518 reflections with *I* > 2σ(*I*)
Graphite monochromator	*R*_int_ = 0.065
ω scans	θ_max_ = 68.2°, θ_min_ = 3.8°
Absorption correction: multi-scan (*ABSCOR*; Higashi, 2001)	*h* = −14→14
*T*_min_ = 0.764, *T*_max_ = 1.000	*k* = −14→14
20279 measured reflections	*l* = −16→16

### Refinement

**Table:**

Refinement on *F*^2^	Secondary atom site location: difference Fourier map
Least-squares matrix: full	Hydrogen site location: inferred from neighbouring sites
*R*[*F*^2^ > 2σ(*F*^2^)] = 0.068	H-atom parameters constrained
*wR*(*F*^2^) = 0.198	*w* = 1/[σ^2^(*F*_o_^2^) + (0.0991*P*)^2^] where *P* = (*F*_o_^2^ + 2*F*_c_^2^)/3
*S* = 0.98	(Δ/σ)_max_ < 0.001
6348 reflections	Δρ_max_ = 0.71 e Å^−^^3^
469 parameters	Δρ_min_ = −0.36 e Å^−^^3^
0 restraints	Extinction correction: *SHELXL97* (Sheldrick, 2008), Fc^*^=kFc[1+0.001xFc^2^λ^3^/sin(2θ)]^-1/4^
Primary atom site location: structure-invariant direct methods	Extinction coefficient: 0.0038 (5)

### Special details

**Table:**

Geometry. All e.s.d.'s (except the e.s.d. in the dihedral angle between two l.s. planes) are estimated using the full covariance matrix. The cell e.s.d.'s are taken into account individually in the estimation of e.s.d.'s in distances, angles and torsion angles; correlations between e.s.d.'s in cell parameters are only used when they are defined by crystal symmetry. An approximate (isotropic) treatment of cell e.s.d.'s is used for estimating e.s.d.'s involving l.s. planes.
Refinement. Refinement of *F*^2^ against ALL reflections. The weighted *R*-factor *wR* and goodness of fit *S* are based on *F*^2^, conventional *R*-factors *R* are based on *F*, with *F* set to zero for negative *F*^2^. The threshold expression of *F*^2^ > σ(*F*^2^) is used only for calculating *R*-factors(gt) *etc*. and is not relevant to the choice of reflections for refinement. *R*-factors based on *F*^2^ are statistically about twice as large as those based on *F*, and *R*- factors based on ALL data will be even larger.

### Fractional atomic coordinates and isotropic or equivalent isotropic displacement parameters (Å^2^)

**Table:**

	*x*	*y*	*z*	*U*_iso_*/*U*_eq_	
Fe1	0.0000	0.0000	0.0000	0.0631 (3)	
N1	−0.0906 (3)	0.1372 (3)	0.0160 (2)	0.0632 (8)	
N2	0.1223 (3)	0.0411 (3)	0.0499 (2)	0.0607 (8)	
N3	0.1490 (3)	0.1625 (3)	−0.2526 (3)	0.0708 (9)	
C1	−0.2519 (3)	0.1171 (3)	−0.0458 (3)	0.0642 (10)	
H1	−0.3217	0.1499	−0.0597	0.077*	
C2	−0.1919 (4)	0.1733 (3)	−0.0101 (3)	0.0633 (10)	
C3	−0.2255 (4)	0.2805 (3)	0.0038 (3)	0.0626 (10)	
C4	−0.3147 (4)	0.3540 (3)	−0.0154 (3)	0.0700 (11)	
H2	−0.3725	0.3357	−0.0397	0.084*	
C5	−0.3175 (4)	0.4548 (3)	0.0017 (3)	0.0727 (11)	
H3	−0.3770	0.5064	−0.0119	0.087*	
C6	−0.2325 (4)	0.4806 (3)	0.0392 (3)	0.0708 (11)	
H4	−0.2353	0.5498	0.0501	0.085*	
C7	−0.1459 (4)	0.4077 (3)	0.0602 (3)	0.0647 (10)	
H5	−0.0904	0.4247	0.0876	0.078*	
C8	−0.1409 (3)	0.3070 (3)	0.0403 (3)	0.0614 (10)	
C9	−0.0590 (4)	0.2171 (3)	0.0474 (3)	0.0633 (10)	
C10	0.0401 (4)	0.2143 (3)	0.0756 (3)	0.0652 (10)	
H6	0.0517	0.2740	0.0965	0.078*	
C11	0.1248 (3)	0.1345 (3)	0.0770 (3)	0.0641 (10)	
C12	0.2283 (4)	0.1355 (3)	0.1073 (3)	0.0659 (10)	
C13	0.2707 (4)	0.2092 (4)	0.1409 (3)	0.0710 (11)	
H7	0.2286	0.2739	0.1484	0.085*	
C14	0.3761 (4)	0.1848 (4)	0.1630 (3)	0.0770 (12)	
H8	0.4072	0.2346	0.1847	0.092*	
C15	0.4377 (4)	0.0903 (4)	0.1544 (3)	0.0743 (11)	
H9	0.5101	0.0762	0.1699	0.089*	
C16	0.3938 (4)	0.0151 (4)	0.1230 (3)	0.0713 (11)	
H10	0.4346	−0.0508	0.1180	0.086*	
C17	0.2886 (4)	0.0396 (3)	0.0992 (3)	0.0663 (10)	
C18	0.2207 (4)	−0.0179 (3)	0.0635 (3)	0.0628 (10)	
C19	0.0970 (3)	0.0993 (3)	−0.1600 (3)	0.0612 (10)	
P1	0.58963 (9)	0.29255 (9)	0.46006 (8)	0.0668 (3)	
C20	0.4674 (4)	0.3415 (3)	0.4124 (3)	0.0688 (11)	
C21	0.4919 (4)	0.3757 (4)	0.2970 (4)	0.0797 (12)	
H11	0.5736	0.3774	0.2446	0.096*	
C22	0.3987 (5)	0.4070 (4)	0.2584 (4)	0.0918 (14)	
H12	0.4169	0.4307	0.1794	0.110*	
C23	0.2798 (5)	0.4047 (4)	0.3323 (4)	0.0914 (14)	
H13	0.2159	0.4245	0.3047	0.110*	
C24	0.2530 (4)	0.3735 (4)	0.4472 (4)	0.0896 (14)	
H14	0.1712	0.3732	0.4986	0.107*	
C25	0.3463 (4)	0.3429 (3)	0.4864 (4)	0.0777 (12)	
H15	0.3281	0.3224	0.5650	0.093*	
C26	0.5437 (3)	0.2790 (3)	0.6049 (3)	0.0636 (10)	
C27	0.4390 (3)	0.2088 (3)	0.6914 (3)	0.0686 (10)	
H16	0.3935	0.1653	0.6743	0.082*	
C28	0.4017 (4)	0.2025 (3)	0.8011 (3)	0.0689 (10)	
H17	0.3278	0.1582	0.8587	0.083*	
C29	0.4711 (4)	0.2603 (3)	0.8277 (3)	0.0732 (11)	
H18	0.4453	0.2547	0.9040	0.088*	
C30	0.5782 (4)	0.3266 (3)	0.7441 (3)	0.0753 (11)	
H19	0.6273	0.3644	0.7631	0.090*	
C31	0.6130 (4)	0.3371 (3)	0.6319 (3)	0.0694 (11)	
H20	0.6847	0.3844	0.5736	0.083*	
C32	0.7236 (4)	0.3919 (3)	0.3697 (3)	0.0646 (10)	
C33	0.7121 (4)	0.5078 (4)	0.3189 (3)	0.0752 (11)	
H21	0.6330	0.5348	0.3281	0.090*	
C34	0.8169 (4)	0.5832 (4)	0.2549 (3)	0.0814 (13)	
H22	0.8098	0.6621	0.2191	0.098*	
C35	0.9311 (4)	0.5443 (4)	0.2431 (3)	0.0826 (13)	
H23	1.0023	0.5969	0.2004	0.099*	
C36	0.9432 (4)	0.4301 (4)	0.2925 (3)	0.0857 (13)	
H24	1.0225	0.4035	0.2831	0.103*	
C37	0.8384 (4)	0.3537 (4)	0.3563 (3)	0.0746 (11)	
H25	0.8462	0.2747	0.3909	0.090*	
C38	0.6318 (4)	0.1582 (3)	0.4509 (3)	0.0679 (10)	
C39	0.6386 (4)	0.0666 (4)	0.5426 (3)	0.0843 (13)	
H26	0.6145	0.0719	0.6146	0.101*	
C40	0.6812 (4)	−0.0337 (4)	0.5290 (4)	0.0928 (14)	
H27	0.6843	−0.0975	0.5925	0.111*	
C41	0.7186 (4)	−0.0409 (4)	0.4251 (3)	0.0811 (12)	
H28	0.7492	−0.1091	0.4164	0.097*	
C42	0.7123 (4)	0.0496 (4)	0.3336 (4)	0.0839 (13)	
H29	0.7381	0.0439	0.2617	0.101*	
C43	0.6688 (4)	0.1485 (4)	0.3452 (3)	0.0785 (12)	
H30	0.6637	0.2107	0.2816	0.094*	
C44	0.9787 (5)	0.1696 (6)	0.5651 (5)	0.119 (2)	
C45	0.9684 (5)	0.0429 (5)	0.6275 (4)	0.129 (2)	
H31	1.0248	0.0088	0.5762	0.193*	
H32	0.8837	0.0120	0.6538	0.193*	
H33	0.9901	0.0251	0.6933	0.193*	
C46	0.9225 (5)	0.2432 (6)	0.6308 (5)	0.141 (2)	
H34	0.9285	0.3222	0.5776	0.212*	
H35	0.9665	0.2393	0.6790	0.212*	
H36	0.8356	0.2163	0.6789	0.212*	
O1	1.0386 (3)	0.2122 (3)	0.4591 (3)	0.1390 (15)	

### Atomic displacement parameters (Å^2^)

**Table:**

	*U*^11^	*U*^22^	*U*^33^	*U*^12^	*U*^13^	*U*^23^
Fe1	0.0676 (6)	0.0566 (6)	0.0485 (5)	−0.0194 (4)	−0.0090 (4)	−0.0133 (4)
N1	0.071 (2)	0.0558 (19)	0.0446 (16)	−0.0205 (16)	−0.0090 (15)	−0.0100 (14)
N2	0.0626 (19)	0.0485 (18)	0.0501 (16)	−0.0144 (15)	−0.0066 (15)	−0.0106 (14)
N3	0.077 (2)	0.062 (2)	0.0551 (18)	−0.0193 (17)	−0.0078 (17)	−0.0175 (16)
C1	0.066 (2)	0.059 (2)	0.059 (2)	−0.0122 (19)	−0.0180 (19)	−0.0174 (19)
C2	0.070 (2)	0.058 (2)	0.0459 (19)	−0.013 (2)	−0.0130 (18)	−0.0115 (18)
C3	0.071 (2)	0.053 (2)	0.0461 (19)	−0.017 (2)	−0.0084 (18)	−0.0117 (17)
C4	0.079 (3)	0.063 (3)	0.057 (2)	−0.008 (2)	−0.020 (2)	−0.016 (2)
C5	0.081 (3)	0.061 (3)	0.065 (2)	−0.004 (2)	−0.021 (2)	−0.021 (2)
C6	0.085 (3)	0.059 (3)	0.056 (2)	−0.013 (2)	−0.016 (2)	−0.0188 (19)
C7	0.074 (2)	0.059 (2)	0.047 (2)	−0.018 (2)	−0.0132 (18)	−0.0128 (18)
C8	0.067 (2)	0.052 (2)	0.0452 (19)	−0.0191 (19)	−0.0066 (17)	−0.0106 (17)
C9	0.068 (2)	0.058 (2)	0.048 (2)	−0.021 (2)	−0.0099 (18)	−0.0124 (18)
C10	0.072 (3)	0.056 (2)	0.054 (2)	−0.017 (2)	−0.0136 (19)	−0.0151 (18)
C11	0.062 (2)	0.060 (2)	0.054 (2)	−0.017 (2)	−0.0110 (18)	−0.0135 (19)
C12	0.070 (2)	0.061 (3)	0.049 (2)	−0.019 (2)	−0.0112 (18)	−0.0125 (19)
C13	0.078 (3)	0.067 (3)	0.057 (2)	−0.013 (2)	−0.019 (2)	−0.017 (2)
C14	0.092 (3)	0.063 (3)	0.065 (2)	−0.019 (2)	−0.022 (2)	−0.018 (2)
C15	0.082 (3)	0.070 (3)	0.061 (2)	−0.018 (2)	−0.023 (2)	−0.016 (2)
C16	0.079 (3)	0.063 (3)	0.060 (2)	−0.013 (2)	−0.019 (2)	−0.017 (2)
C17	0.070 (3)	0.059 (2)	0.052 (2)	−0.017 (2)	−0.0132 (19)	−0.0105 (18)
C18	0.071 (2)	0.055 (2)	0.048 (2)	−0.016 (2)	−0.0112 (18)	−0.0154 (18)
C19	0.065 (2)	0.055 (2)	0.057 (2)	−0.0157 (18)	−0.0117 (19)	−0.0239 (19)
P1	0.0737 (7)	0.0586 (6)	0.0496 (6)	−0.0164 (5)	−0.0083 (5)	−0.0145 (5)
C20	0.074 (3)	0.058 (2)	0.057 (2)	−0.011 (2)	−0.014 (2)	−0.0137 (19)
C21	0.088 (3)	0.074 (3)	0.069 (3)	−0.006 (2)	−0.025 (2)	−0.024 (2)
C22	0.111 (4)	0.083 (3)	0.079 (3)	−0.010 (3)	−0.040 (3)	−0.024 (3)
C23	0.097 (4)	0.074 (3)	0.093 (4)	−0.018 (3)	−0.042 (3)	−0.013 (3)
C24	0.081 (3)	0.071 (3)	0.093 (3)	−0.017 (2)	−0.028 (3)	−0.010 (3)
C25	0.078 (3)	0.065 (3)	0.067 (3)	−0.021 (2)	−0.015 (2)	−0.010 (2)
C26	0.069 (2)	0.057 (2)	0.051 (2)	−0.0120 (19)	−0.0112 (18)	−0.0165 (17)
C27	0.075 (2)	0.062 (2)	0.055 (2)	−0.017 (2)	−0.0120 (19)	−0.0177 (19)
C28	0.072 (2)	0.062 (2)	0.053 (2)	−0.008 (2)	−0.0110 (19)	−0.0146 (19)
C29	0.087 (3)	0.062 (3)	0.057 (2)	−0.010 (2)	−0.017 (2)	−0.0183 (19)
C30	0.086 (3)	0.069 (3)	0.061 (2)	−0.016 (2)	−0.018 (2)	−0.022 (2)
C31	0.071 (2)	0.062 (2)	0.058 (2)	−0.014 (2)	−0.0109 (19)	−0.0171 (19)
C32	0.069 (2)	0.061 (2)	0.050 (2)	−0.016 (2)	−0.0108 (19)	−0.0164 (18)
C33	0.081 (3)	0.067 (3)	0.060 (2)	−0.018 (2)	−0.008 (2)	−0.022 (2)
C34	0.099 (3)	0.062 (3)	0.061 (2)	−0.026 (3)	−0.008 (2)	−0.019 (2)
C35	0.079 (3)	0.085 (3)	0.055 (2)	−0.035 (3)	−0.004 (2)	−0.015 (2)
C36	0.075 (3)	0.095 (4)	0.064 (3)	−0.019 (3)	−0.013 (2)	−0.017 (2)
C37	0.076 (3)	0.071 (3)	0.060 (2)	−0.011 (2)	−0.020 (2)	−0.012 (2)
C38	0.075 (2)	0.052 (2)	0.056 (2)	−0.0189 (19)	−0.0066 (19)	−0.0147 (19)
C39	0.110 (4)	0.068 (3)	0.057 (2)	−0.002 (3)	−0.017 (2)	−0.020 (2)
C40	0.124 (4)	0.062 (3)	0.063 (3)	−0.001 (3)	−0.014 (3)	−0.016 (2)
C41	0.089 (3)	0.065 (3)	0.061 (3)	−0.015 (2)	−0.004 (2)	−0.018 (2)
C42	0.098 (3)	0.071 (3)	0.069 (3)	−0.005 (3)	−0.016 (2)	−0.028 (2)
C43	0.096 (3)	0.066 (3)	0.058 (2)	−0.006 (2)	−0.016 (2)	−0.019 (2)
C44	0.086 (4)	0.147 (6)	0.086 (4)	−0.038 (4)	−0.034 (3)	0.004 (4)
C45	0.128 (4)	0.127 (5)	0.088 (4)	−0.052 (4)	−0.033 (3)	0.004 (3)
C46	0.096 (4)	0.177 (6)	0.107 (5)	0.018 (4)	−0.020 (4)	−0.033 (5)
O1	0.124 (3)	0.157 (4)	0.083 (3)	−0.039 (3)	−0.029 (2)	0.004 (2)

### Geometric parameters (Å, º)

**Table:**

Fe1—C19^i^	1.971 (4)	C22—C23	1.372 (6)
Fe1—C19	1.971 (4)	C22—H12	0.9500
Fe1—N2	2.008 (3)	C23—C24	1.386 (6)
Fe1—N2^i^	2.008 (3)	C23—H13	0.9500
Fe1—N1^i^	2.023 (3)	C24—C25	1.380 (6)
Fe1—N1	2.023 (3)	C24—H14	0.9500
N1—C9	1.380 (4)	C25—H15	0.9500
N1—C2	1.380 (5)	C26—C31	1.384 (5)
N2—C18	1.373 (5)	C26—C27	1.397 (4)
N2—C11	1.387 (4)	C27—C28	1.375 (5)
N3—C19	1.154 (4)	C27—H16	0.9500
C1—C2	1.377 (5)	C28—C29	1.378 (5)
C1—C18^i^	1.378 (5)	C28—H17	0.9500
C1—H1	0.9500	C29—C30	1.387 (5)
C2—C3	1.460 (5)	C29—H18	0.9500
C3—C4	1.392 (5)	C30—C31	1.394 (5)
C3—C8	1.398 (5)	C30—H19	0.9500
C4—C5	1.392 (5)	C31—H20	0.9500
C4—H2	0.9500	C32—C37	1.374 (5)
C5—C6	1.408 (5)	C32—C33	1.393 (5)
C5—H3	0.9500	C33—C34	1.385 (5)
C6—C7	1.371 (5)	C33—H21	0.9500
C6—H4	0.9500	C34—C35	1.372 (6)
C7—C8	1.408 (5)	C34—H22	0.9500
C7—H5	0.9500	C35—C36	1.375 (6)
C8—C9	1.443 (5)	C35—H23	0.9500
C9—C10	1.368 (5)	C36—C37	1.391 (5)
C10—C11	1.373 (5)	C36—H24	0.9500
C10—H6	0.9500	C37—H25	0.9500
C11—C12	1.445 (5)	C38—C39	1.379 (5)
C12—C17	1.389 (5)	C38—C43	1.404 (5)
C12—C13	1.397 (5)	C39—C40	1.394 (5)
C13—C14	1.383 (5)	C39—H26	0.9500
C13—H7	0.9500	C40—C41	1.366 (5)
C14—C15	1.382 (6)	C40—H27	0.9500
C14—H8	0.9500	C41—C42	1.368 (6)
C15—C16	1.400 (5)	C41—H28	0.9500
C15—H9	0.9500	C42—C43	1.369 (5)
C16—C17	1.392 (5)	C42—H29	0.9500
C16—H10	0.9500	C43—H30	0.9500
C17—C18	1.454 (5)	C44—O1	1.255 (6)
C18—C1^i^	1.378 (5)	C44—C46	1.498 (8)
P1—C38	1.788 (4)	C44—C45	1.499 (8)
P1—C20	1.792 (4)	C45—H31	0.9800
P1—C32	1.795 (4)	C45—H32	0.9800
P1—C26	1.797 (4)	C45—H33	0.9800
C20—C21	1.392 (5)	C46—H34	0.9800
C20—C25	1.401 (5)	C46—H35	0.9800
C21—C22	1.376 (6)	C46—H36	0.9800
C21—H11	0.9500		
			
C19^i^—Fe1—C19	180.0	C22—C21—C20	120.4 (4)
C19^i^—Fe1—N2	89.10 (12)	C22—C21—H11	119.8
C19—Fe1—N2	90.90 (12)	C20—C21—H11	119.8
C19^i^—Fe1—N2^i^	90.90 (12)	C23—C22—C21	121.1 (4)
C19—Fe1—N2^i^	89.10 (12)	C23—C22—H12	119.5
N2—Fe1—N2^i^	180.0	C21—C22—H12	119.5
C19^i^—Fe1—N1^i^	87.27 (13)	C22—C23—C24	119.8 (5)
C19—Fe1—N1^i^	92.73 (13)	C22—C23—H13	120.1
N2—Fe1—N1^i^	90.33 (13)	C24—C23—H13	120.1
N2^i^—Fe1—N1^i^	89.67 (13)	C25—C24—C23	119.4 (5)
C19^i^—Fe1—N1	92.73 (13)	C25—C24—H14	120.3
C19—Fe1—N1	87.27 (13)	C23—C24—H14	120.3
N2—Fe1—N1	89.67 (13)	C24—C25—C20	121.2 (4)
N2^i^—Fe1—N1	90.33 (13)	C24—C25—H15	119.4
N1^i^—Fe1—N1	180.0	C20—C25—H15	119.4
C9—N1—C2	106.4 (3)	C31—C26—C27	119.4 (3)
C9—N1—Fe1	127.1 (3)	C31—C26—P1	120.1 (3)
C2—N1—Fe1	126.4 (3)	C27—C26—P1	120.5 (3)
C18—N2—C11	106.3 (3)	C28—C27—C26	120.1 (4)
C18—N2—Fe1	126.6 (2)	C28—C27—H16	119.9
C11—N2—Fe1	127.1 (3)	C26—C27—H16	119.9
C2—C1—C18^i^	126.4 (4)	C27—C28—C29	120.2 (4)
C2—C1—H1	116.8	C27—C28—H17	119.9
C18^i^—C1—H1	116.8	C29—C28—H17	119.9
C1—C2—N1	124.8 (4)	C28—C29—C30	120.5 (4)
C1—C2—C3	124.7 (4)	C28—C29—H18	119.7
N1—C2—C3	110.5 (3)	C30—C29—H18	119.7
C4—C3—C8	120.7 (4)	C29—C30—C31	119.2 (4)
C4—C3—C2	133.6 (4)	C29—C30—H19	120.4
C8—C3—C2	105.6 (4)	C31—C30—H19	120.4
C5—C4—C3	118.7 (4)	C26—C31—C30	120.4 (3)
C5—C4—H2	120.7	C26—C31—H20	119.8
C3—C4—H2	120.7	C30—C31—H20	119.8
C4—C5—C6	120.3 (4)	C37—C32—C33	119.9 (4)
C4—C5—H3	119.8	C37—C32—P1	119.3 (3)
C6—C5—H3	119.8	C33—C32—P1	120.7 (3)
C7—C6—C5	121.3 (4)	C34—C33—C32	119.4 (4)
C7—C6—H4	119.4	C34—C33—H21	120.3
C5—C6—H4	119.4	C32—C33—H21	120.3
C6—C7—C8	118.5 (4)	C35—C34—C33	120.3 (4)
C6—C7—H5	120.8	C35—C34—H22	119.9
C8—C7—H5	120.8	C33—C34—H22	119.9
C3—C8—C7	120.5 (4)	C34—C35—C36	120.6 (4)
C3—C8—C9	106.9 (3)	C34—C35—H23	119.7
C7—C8—C9	132.6 (4)	C36—C35—H23	119.7
C10—C9—N1	124.5 (4)	C35—C36—C37	119.5 (4)
C10—C9—C8	124.9 (4)	C35—C36—H24	120.3
N1—C9—C8	110.5 (3)	C37—C36—H24	120.3
C9—C10—C11	127.1 (4)	C32—C37—C36	120.4 (4)
C9—C10—H6	116.5	C32—C37—H25	119.8
C11—C10—H6	116.5	C36—C37—H25	119.8
C10—C11—N2	124.6 (4)	C39—C38—C43	119.1 (4)
C10—C11—C12	124.9 (4)	C39—C38—P1	121.5 (3)
N2—C11—C12	110.6 (4)	C43—C38—P1	119.2 (3)
C17—C12—C13	120.9 (4)	C38—C39—C40	119.5 (4)
C17—C12—C11	106.2 (4)	C38—C39—H26	120.2
C13—C12—C11	132.9 (4)	C40—C39—H26	120.2
C14—C13—C12	117.7 (4)	C41—C40—C39	120.5 (4)
C14—C13—H7	121.2	C41—C40—H27	119.8
C12—C13—H7	121.2	C39—C40—H27	119.8
C15—C14—C13	122.1 (4)	C40—C41—C42	120.3 (4)
C15—C14—H8	119.0	C40—C41—H28	119.8
C13—C14—H8	119.0	C42—C41—H28	119.8
C14—C15—C16	120.2 (4)	C41—C42—C43	120.3 (4)
C14—C15—H9	119.9	C41—C42—H29	119.8
C16—C15—H9	119.9	C43—C42—H29	119.8
C17—C16—C15	118.2 (4)	C42—C43—C38	120.3 (4)
C17—C16—H10	120.9	C42—C43—H30	119.9
C15—C16—H10	120.9	C38—C43—H30	119.9
C12—C17—C16	120.9 (4)	O1—C44—C46	121.3 (6)
C12—C17—C18	106.7 (4)	O1—C44—C45	119.2 (7)
C16—C17—C18	132.4 (4)	C46—C44—C45	119.5 (5)
N2—C18—C1^i^	125.4 (4)	C44—C45—H31	109.5
N2—C18—C17	110.2 (3)	C44—C45—H32	109.5
C1^i^—C18—C17	124.4 (4)	H31—C45—H32	109.5
N3—C19—Fe1	175.8 (3)	C44—C45—H33	109.5
C38—P1—C20	110.89 (19)	H31—C45—H33	109.5
C38—P1—C32	107.17 (18)	H32—C45—H33	109.5
C20—P1—C32	109.48 (18)	C44—C46—H34	109.5
C38—P1—C26	109.39 (18)	C44—C46—H35	109.5
C20—P1—C26	110.28 (18)	H34—C46—H35	109.5
C32—P1—C26	109.57 (17)	C44—C46—H36	109.5
C21—C20—C25	118.0 (4)	H34—C46—H36	109.5
C21—C20—P1	118.8 (3)	H35—C46—H36	109.5
C25—C20—P1	123.1 (3)		

Symmetry code: (i) −*x*, −*y*, −*z*.

### Hydrogen-bond geometry (Å, º)

*Cg*1, *Cg*2, *Cg*3 and *Cg*4 are the centroids of the N2/C11/C12/C17/C18, C12–C17, C26–C31 and N1/C2/C3/C8/C9 rings, respectively.

**Table:**

*D*—H···*A*	*D*—H	H···*A*	*D*···*A*	*D*—H···*A*
C25—H15···N3^ii^	0.95	2.74	3.404 (4)	127
C27—H16···N3^ii^	0.95	2.66	3.259 (5)	121
C34—H22···N3^iii^	0.95	2.57	3.292 (7)	133
C41—H28···N3^iv^	0.95	2.45	3.181 (7)	134
C28—H17···*Cg*1^ii^	0.95	2.63	3.446 (4)	145
C29—H18···*Cg*2^ii^	0.95	2.76	3.582 (4)	145
C33—H21···*Cg*3^v^	0.95	2.90	3.729 (5)	147
C35—H23···*Cg*4^iii^	0.95	2.92	3.733 (4)	145
C45—H32···*Cg*2^vi^	0.98	2.99	3.798 (6)	141
C45—H33···*Cg*1^vi^	0.98	2.87	3.469 (5)	120

Symmetry codes: (ii) *x*, *y*, *z*+1; (iii) −*x*+1, −*y*+1, −*z*; (iv) −*x*+1, −*y*, −*z*; (v) −*x*+1, −*y*+1, −*z*+1; (vi) −*x*+1, −*y*, −*z*+1.
