# Supplementary figures and images for: Crystal structure of bis­(tetra­phenyl­phospho­nium) bis­(cyanido-κC)(29H,31H-tetra­benzo[b,g,l,q]porphinato-κ4 N 29,N 30,N 31,N 32)ferrate(II) acetone disolvate
Source: Acta Crystallogr E Crystallogr Commun. 2015 Jan 31;71(Pt 2):m48–9. doi: 10.1107/S2056989015001735 (PMC4384569; doi:10.1107/S2056989015001735)

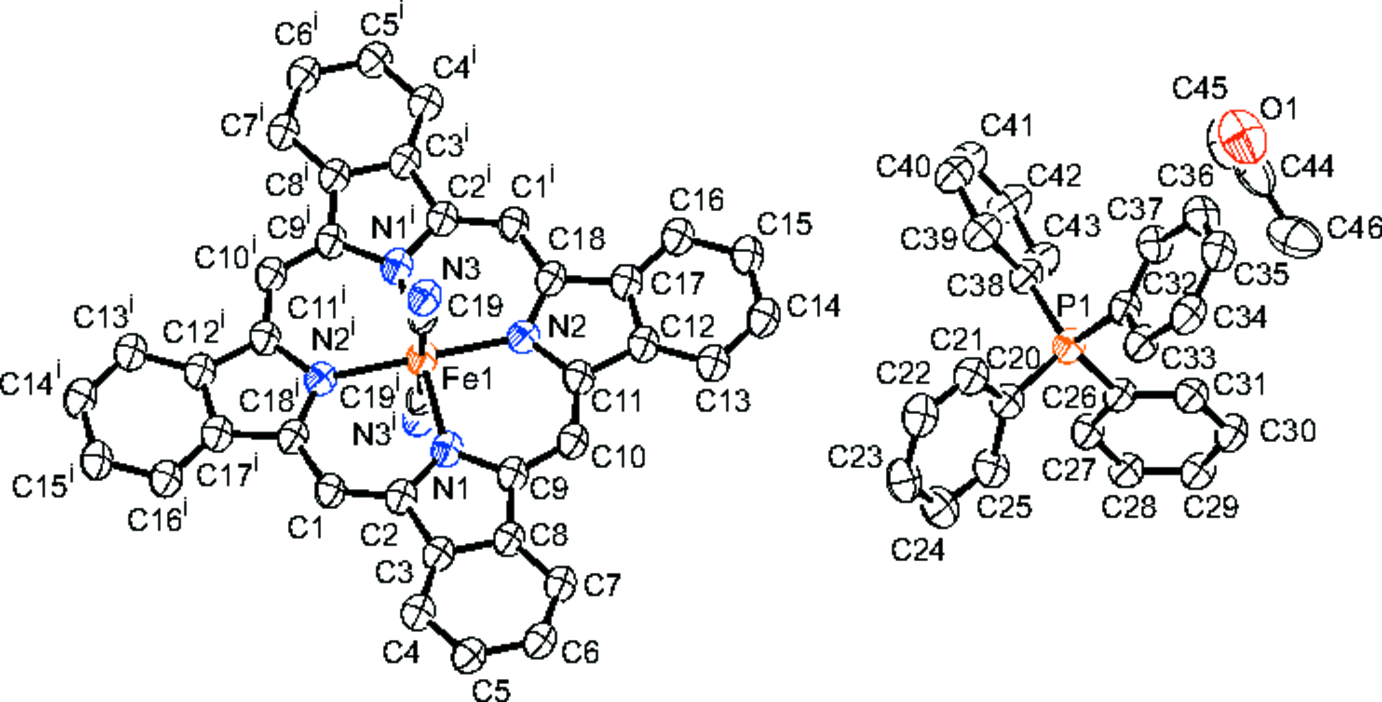

Supplement: Supplementary file 3 [file e-71-00m48-fig1.tif]

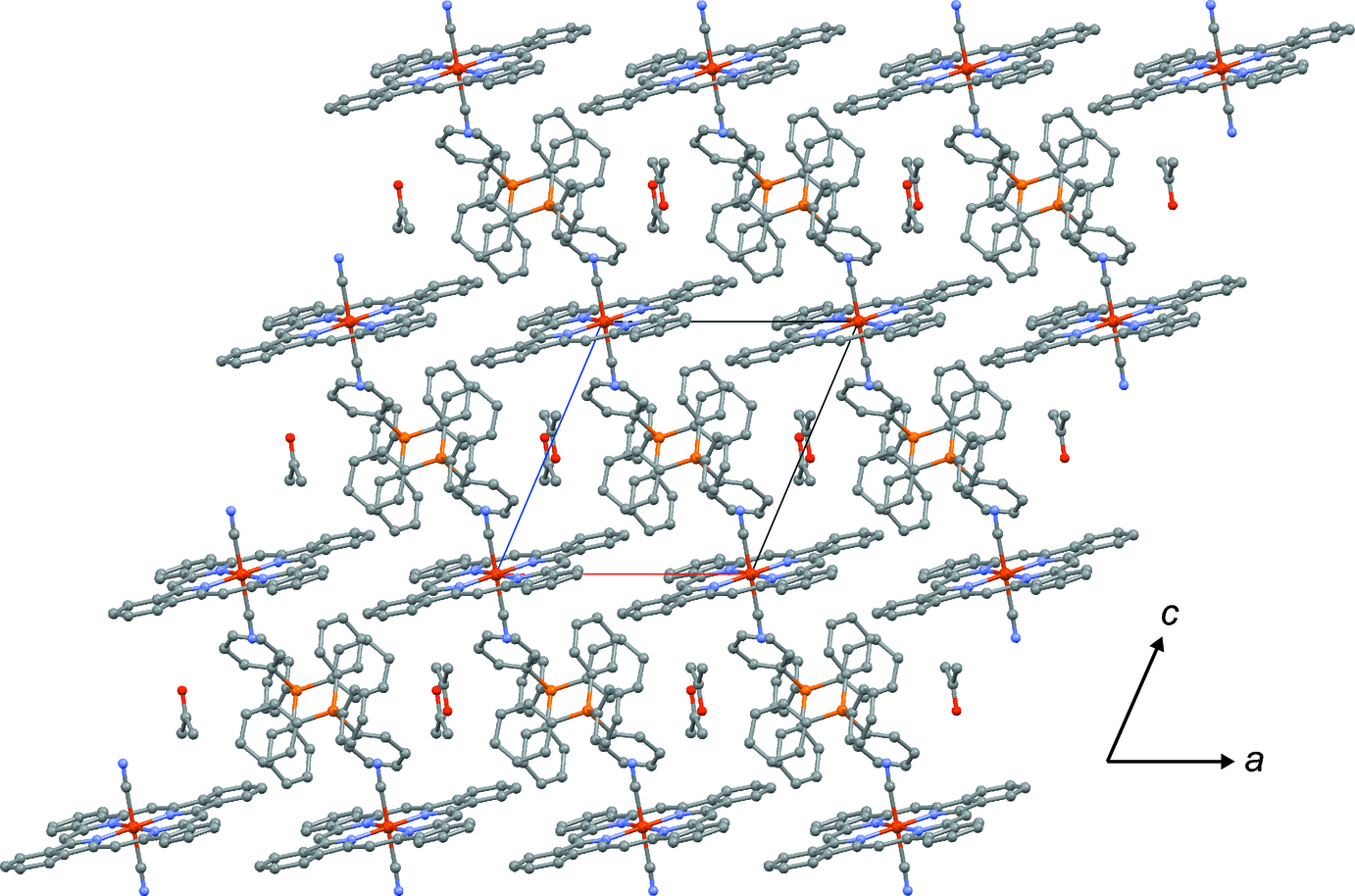

Supplement: Supplementary file 4 [file e-71-00m48-fig2.tif]
